# Comparing Preoperative Anxiety Effects of Brachial Plexus Block and General Anesthesia for Orthopedic Upper-Extremity Surgery: A Randomized, Controlled Trial

**DOI:** 10.3390/medicina58091296

**Published:** 2022-09-16

**Authors:** Gokhan Sertcakacilar, Gunes Ozlem Yildiz, Berhan Bayram, Yaser Pektas, Zafer Cukurova, Gulsum Oya Hergunsel

**Affiliations:** 1 Department of Outcomes Research, Anesthesiology Institute, Cleveland Clinic, Cleveland, OH 44195, USA; 2 Department of Anesthesiology and Reanimation, University of Health Science, Bakırköy Dr. Sadi Konuk Education and Research Hospital, 34147 Istanbul, Turkey; 3 Department of Orthopedic Surgery and Sport Medicine, University of Acıbadem, Acıbadem Altunizade Hospital, 34662 Istanbul, Turkey

**Keywords:** brachial plexus block, general anesthesia, preoperative anxiety, APAIS, orthopedic surgery, upper-extremity surgery

## Abstract

*Background and objectives:* Preoperative anxiety is an enormous feeling of fear that is seen in all patients undergoing surgery. The severity of anxiety may vary depending on the type of surgery and anesthesia to be performed. The aim of this study is to compare the effects of brachial plexus blocks and general anesthesia methods on preoperative anxiety levels in patients who will undergo orthopedic upper-extremity surgery and to determine the factors affecting anxiety. *Materials and Methods:* After randomization, the Amsterdam Preoperative Anxiety and Knowledge Scale (APAIS) questionnaire was applied to the patients to determine the preoperative anxiety level, and then anesthesia was applied according to the anesthesia type determined. Pain scores (1, 8, 16, and 24 h) and total opioid consumption of the patients were recorded postoperatively. *Results:* The APAIS score of the patients in the general anesthesia (GA) group was significantly higher (*p* = 0.021). VAS score medians at 1, 4, and 8 h postoperatively were found to be significantly higher in the GA group (*p* < 0.001, *p* < 0.001 and *p* = 0.044, respectively). *Conclusions:* USG-guided BPB may cause less anxiety than GA in patients who will undergo elective upper-extremity surgery. However, these patients have moderate anxiety, although it is more associated with advanced age, female gender, and education level.

## 1. Introduction

Performing surgical procedures and anesthesia are situations where people do not feel safe due to the current vital risks. Anxiety is a natural reaction that occurs in such situations; it manifests itself with worry and fear. This rate has been reported as being 11–80% in patients in the preoperative period [1]. Increased anxiety before surgery is associated with pathophysiological responses such as hypertension and dysrhythmia [2]. In addition, it is known that intense preoperative anxiety increases morbidity (surgical wound infection, etc.), the need of anesthetic drugs, and postoperative analgesic requirements [3,4,5].

For this reason, many questionnaire studies have been conducted to measure the degree of preoperative anxiety and to reveal its causes [6,7,8]. Apart from the STAI test, which was widely used in previous years, the “Amsterdam Preoperative Anxiety and Information Scale (APAIS)” test, which is more straightforward and takes less time to apply, has also been frequently preferred. The APAIS test has two parts that measure the anxiety and need for information about anesthesia and surgery. The APAIS-A (anxiety) part is the test that measures strain and shows whether the patient has anxiety about anesthesia or surgery. The APAIS-I (information) part estimates whether the person needs information. The usability of this test and its correlation with STAI were investigated [9,10]. A study reported that the correlation of the STAI score with the APAIS-A anxiety score was 0.74, and its correlation with the APAIS-I information acquisition score was 0.16 [9]. In addition, the researchers concluded that the APAIS scale could be used as safely as the STAI test in preoperative anxiety measurement.

Different anesthesia methods are successfully performed in orthopedic upper-extremity surgery. With the widespread use of ultrasound in daily anesthesia practice, peripheral nerve blocks have also been used frequently as a method of anesthesia. The anesthesia method performed independently of the surgical procedure may also affect the preoperative anxiety level. In the literature, the effects of general and spinal anesthesia techniques on preoperative anxiety levels have been compared for some specific surgical methods such as c-sections and perianal region surgeries [11,12]. However, no study has been reported between peripheral nerve block and general anesthesia techniques.

In this study, it was aimed to compare the effects of brachial plexus blocks and general anesthesia methods on preoperative anxiety levels in patients who will undergo upper-extremity surgery and to determine the factors affecting these scores.

## 2. Materials and Methods

### 2.1. Study Design and Patient Selection

This prospective randomized controlled trial was conducted after obtaining approval (2020/36) from the ethics committee of a training and research hospital. The study included one hundred twenty patients whose ASA scores are I-II-III, aged 18–75 years, who would undergo upper-extremity surgery at the orthopedics clinic under elective conditions. Before enrolling in the study, all participants were informed about the study, both anesthesia methods that could be applied were explained, and a consent form was signed. Patients with ASA ≥ 4, morbid obesity (BMI ≥ 35), difficulty reading and understanding the anxiety form, mental illness, illiteracy, psychiatric disease, and a condition that prevents peripheral nerve block (neurological deficit, local anesthetic allergy, peripheral vascular disease, and coagulopathy) were excluded from the study. The study was carried out in accordance with the principles set out in the 1995 Declaration of Helsinki (as revised in Brazil, 2013). The study was registered at clinicaltrials.gov (NCT05476198) and followed the Consolidated Standards of Reporting Trials (CONSORT) guidelines.

The sample size was calculated by the pilot study. Power analysis was performed according to the primary outcome of the study, the preoperative anxiety score. Twenty patients were assigned to each group to determine the minimum sample size. The mean APAIS score obtained in the pilot study was 16.69 ± 4.32 in Group GA and 12.76 ± 6.12 in Group BPB. Accordingly, at least 49 participants were calculated for each group, with an effect size of 0.741, α error = 0.05, and 95% power. Considering a possible dropout rate of 20%, 60 patients for each group were included in the study. The G *Power 3.1.9.2 program was used to calculate the sample size of the study.

### 2.2. Determination of Preoperative Anxiety Levels

On the day of surgery, patients were randomized to one of the GA and BPB groups by a sealed envelope method by an anesthesiology nurse (DÖ) who was not included in the study. After the anesthesia method was explained to the patient, the anxiety questionnaire was administered by an anesthesia nurse who was blind to the study. The Amsterdam Preoperative Anxiety and Knowledge Scale (APAIS), which consists of six statements, was used for the study. In the APAIS questionnaire, the source of concern is evaluated on three scales: the patient’s anxiety about anesthesia or surgery and the anxiety caused by a lack of information. The APAIS questionnaire includes six statements for these three sources to assess anxiety. In the questionnaire, each statement is given a numerical value based on a 5-point Likert scale; these values range from 1–5: 1 = not at all, 2 = slightly, 3 = moderately, 4 = quite a bit, and 5 = extremely.

Anesthesia anxiety is calculated by the scores on questions 1 and 2 and surgical anxiety on questions 4 and 5. The statements expressing the desire to obtain information about anesthesia and surgery are questions 3 and 6. The lowest score is six, and the highest score is 30. In addition, after the APAIS questionnaire, patients were presented with nine statements indicating the estimated reason for their anxiety about anesthesia [13]. They were asked to choose the statements that fit them.

### 2.3. Monitoring and Preparation

After the questionnaire, the patients were taken to the operating room. Before anesthesia induction, patients were monitored with 3-lead ECGs, noninvasive blood pressure cuffs, and pulse oximetry probes. Nurse anesthetists followed the patient’s ECG, heart rate (HR), peripheral oxygen saturation (SpO2) momentarily, and systolic and diastolic blood pressures at 5 min intervals throughout the surgery. The anesthesiologists inserted a 20-gauge intravenous cannula from the dorsal side of the hand in the nonoperated extremity and, after that, started the infusion of lactated Ringer solution at a dose of 4–8 mL/kg/hour. All patients were premedicated with 0.03–0.05 mg/kg midazolam, and the anesthesia method determined by randomization was applied.

### 2.4. General Anesthesia

After premedication, induction was achieved with 2–3 mg/kg propofol, 1–2 mcg/kg fentanyl, and 0.6–0.8 mg/kg rocuronium. After adequate muscle relaxation was achieved, tracheal intubation was performed, and anesthesia was maintained with 0.8–1 MAC sevoflurane and 0.05–0.1 mcg/kg/min remifentanil infusion. At the end of the surgery, 1 gr iv paracetamol and 100 mg tramadol were administered as analgesics. At the end of the surgery, the maintenance of anesthesia was terminated, and the patients were extubated after being decurarized with 0.01 mg/kg atropine and 0.03 mg/kg neostigmine when spontaneous breathing was achieved.

### 2.5. Brachial Plexus Blocks

The patients were positioned according to their determined brachial plexus block type. A General Electric (GE) LOGIQ e model (GE Medical Systems, Phoenix, AZ, USA) was used as USG. The Linear Multifrequency 12L probe was passed through a sterile camera sheath and made suitable for use in the surgical field. Stimuplex A**^®^** (B. Braun Aesculap, Hongo, Bunkyo-ku, Japan) 21G was used as the needle. A needle of 100 mm in length for supraclavicular and infraclavicular block and 50 mm in length for interscalene block was preferred. The entry site of the peripheral nerve block needle was sterilized with an antiseptic solution containing 10% povidone-iodine. After the preparation the patient was informed, and 4cc 2% lidocaine hydrochloride was injected into the entry site to provide local anesthesia. The USG probe was positioned according to the block type, and the in-plane technique was always used during application. A local anesthetic was injected at the required dose and concentration from the appropriate brachial plexus region according to the decided upper-extremity block.

#### 2.5.1. Interscalene Block

Brachial plexus nerve roots at the C6 level were detected by in-plane imaging with a linear ultrasound probe. A needle was advanced from lateral to medial through the anterior and middle scalene muscles towards the nerve roots forming the brachial plexus. After the needle location was confirmed by negative aspiration, 0.25%, 15 mL of bupivacaine hydrochloride was injected around the roots of C5, C6, and C7.

#### 2.5.2. Supraclavicular Block

The transducer was placed over the supraclavicular fossa for the best view of the subclavian artery and brachial plexus while the patient was lying on his/her back facing the opposite side. After the brachial plexus was fixed laterally to the subclavian artery and superficially above the first rib, the needle was advanced in the same plane along the long axis of the transducer using the in-plane technique. Bupivacaine hydrochloride at a concentration of 0.25% was injected around the plexus with a total of 20 mL of intermittent negative aspiration.

#### 2.5.3. Infraclavicular Block

The infraclavicular block was performed according to the lateral sagittal infraclavicular block technique. The USG probe was placed 1 cm before the intersection point between the coracoid process and the clavicle in the sagittal plane. The in-plane technique was used during the application. Bupivacaine hydrochloride at a concentration of 0.25% was injected with intermittent negative aspiration, 20 mL in total, around the posterior, lateral, and medial cord, respectively.

### 2.6. Postoperative Pain Management and Evaluation

Standard orthopedic service analgesic protocol was applied to the patients who underwent general anesthesia in the postoperative period and after the block returned and after the patient started to feel pain in the patients who underwent peripheral block. Accordingly, all patients were given 4 × 30 mg of tramadol and 2 × 20 mg of tenoxicam as standard. A total of 20 mg of meperidine was administered as rescue analgesia. In the postoperative period, the NRS pain score was evaluated at 1, 8, 16, and 24 h for all patients. Pain score follow-ups were performed by anesthesia nurses who were not included in the study and were blind to randomization, just like the anesthesia questionnaire.

### 2.7. Statistical Analysis

The data collected in the study were evaluated with the SPSS 22.00 program for Windows 10. The Kolmogorov–Smirnov test was used to check the normality of the data distributions. For descriptive statistics, categorical variables are given as percentage (%) and numerical variables as mean ± standard deviation and median. In the comparison of the quantitative data of the 2 groups when the normality conditions are met, a two-sample independent *t*-test was used, whereas a chi-square test was used when the variables were qualitative. The Mann–Whitney U test and Kruskall–Wallis test were used for quantitative variable data comparisons where normality conditions were not met. The statistical significance level of alpha was accepted as *p* < 0.05. For further analysis, bivariate Pearson’s correlation analysis was assessed in order to explore the relationship between age and APAIS scores.

## 3. Results

**Figure 1:**
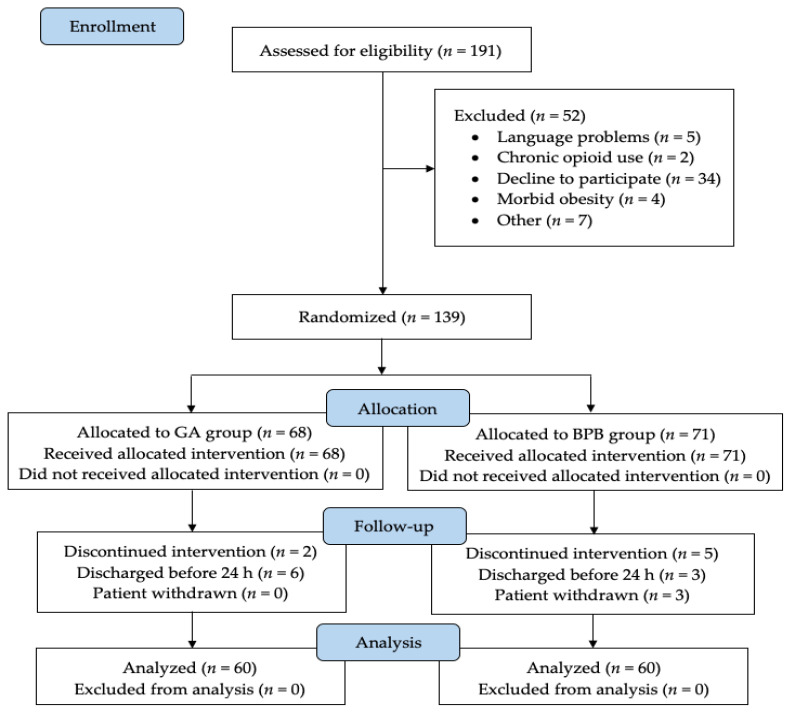
CONSORT flow chart of the study. GA: general anesthesia; BPB: brachial plexus block.

The study was carried out in a training and research hospital affiliated to the university between 5 February 2019 and 15 April 2021. A total of 120 patients between the ages of 18–75 who underwent orthopedic upper-extremity surgery were included in the study (Figure 1). In total, 40.8% (n = 49) of the patients were female and 59.1% (n = 71) were male. A total of 53.3% (n = 64) of the subjects had an ASA score of II, and 69.1% (n = 83) had a high school or higher education level. There was no significant difference between the groups in terms of demographic data (Table 1). The most common type of surgery performed by the patients was ARIF (74.1%, n = 89). Due to the variety of surgeries performed in our hospital, we classified the small portion of the surgeries (9.1%, n = 11) of our patients under the heading of “other”. A total of 66% of the cases (n = 80) were patients who had previously been operated for any reason, and thus received anesthesia.

Of the patients, 44.1% (n = 53) had general anesthesia, 9.1% (n = 16) had regional anesthesia, and 13.3% (n = 11) had both general anesthesia and regional anesthesia. There was no difference between the groups in terms of operation and anesthesia experience. 

When the relationship between anesthesia type and anxiety, which was the primary aim of our study, was investigated, it was found that the patients in the general anesthesia group had a significantly higher APAIS score (*p* = 0.021). While there was no difference between the two groups in terms of information acquisition scores (*p* = 0.429), a statistically significant difference was found between anxiety and total APAIS scores (*p* = 0.007, *p* = 0.021, respectively) (Table 2). Considering the reasons for anxiety, the patients in the general anesthesia group had a significantly higher fear of death, inability to wake up after the operation, and the fear of postoperative pain (*p* < 0.001, *p* = 0.007, and *p* = 0.010, respectively). In patients in the BPB group, operation site incompatibility and fear of needle/interference were found to be statistically higher (*p* = 0.004 and *p* = 0.021, respectively). There was no statistically significant difference between age and desire to obtain information subscores in both groups (r = −0.197; *p* = 0.481, r = −0.066, and *p* = 0.814, respectively) (Table 3). 

In both groups (group GA and BPD), women were found to have higher anxiety levels than men (*p* = 0.001 and *p* = 0.015, respectively). Education levels of both groups were similar. In addition, it was observed that APAIS scores increased significantly as the education level increased in both the GA and BPD groups (*p* = 0.041 and *p* = 0.011, respectively) (Table 4). For postoperative 1-8-16 VAS score medians per hour, it was found to be significantly lower in the regional anesthesia group (*p* < 0.001, *p* < 0.001, and *p* = 0.044, respectively). At the 24th hour, there was no statistically significant relationship between the two groups in terms of VAS scores (*p* = 0.750).

## 4. Discussion

The presented study showed that ultrasound-guided brachial plexus block significantly reduces anxiety levels in patients who will undergo upper-extremity surgery compared with general anesthesia. The brachial plexus block also helped reduce pain and analgesic consumption in this group of patients. In addition, we concluded that it also reduced the length of stay in the hospital, which was consistent with previous studies.

Brachial plexus blocks have been used for many years in orthopedic upper-extremity surgery to provide anesthesia and postoperative analgesia [14]. Many peripheral nerve blocks, such as infraclavicular, supraclavicular, and interscalene blocks, are successfully applied by determining the brachial plexus level according to the surgical site. Especially in the last 20 years, it has been accepted that regional anesthesia methods are more reliable than general anesthesia when appropriate surgical conditions are provided [15]. Peripheral nerve blocks are superior to general anesthesia in terms of features such as being conscious during the operation, no change in respiratory functions, preservation of airway reflexes, and providing analgesia in the postoperative period. Thanks to their postoperative analgesic effect, peripheral nerve blocks can prevent complications such as respiratory depression, urinary retention, nausea, and vomiting due to opioids and enable patients to mobilize in a shorter time [14,16]. However, despite all these advantages, the effect of choosing nerve block as an anesthetic method in a patient with preoperative anxiety on the patient’s anxiety is unknown and has not been investigated.

In our study, we aimed to determine whether the anxiety of patients undergoing orthopedic upper-extremity surgery varies according to general anesthesia and brachial plexus block with the APAIS questionnaire and to choose the possible causes of this situation in patients with anxiety. The APAIS questionnaire consists of two subscore groups: anxiety and desire to obtain information. It is an anxiety measurement tool that allows us to obtain important information about anesthesia and surgery [17]. APAIS is both reliable and practical [18]. The APAIS questionnaire consists of six questions in total, and the questions are simple and understandable. Therefore, it is carried out quickly and without patients’ distraction. It is a widely known and used scale by doctors today [17]. APAIS-A gives the anxiety score, and APAIS-I gives the patients’ desire for information score [19].

In this study, it was determined that the patients in the general anesthesia group had statistically higher anxiety scores than the regional anesthesia group. According to the questions we asked to understand the cause of anxiety, it was determined that this difference in the general anesthesia group was mainly due to thoughts such as not being able to wake up after anesthesia, waking up during surgery, or dying from anesthesia. Patients who underwent brachial plexus block may have lower anxiety levels because they know that they will be awake and that there will be no such risks for them.

The most common causes of anxiety in patients with anesthesia anxiety differed between the groups. The most common cause of anxiety in patients who received general anesthesia was the fear of dying during the operation. In contrast, the most common cause in the regional anesthesia group was pain and suffering during the procedure. In a survey conducted in the literature on knowledge and fears about anesthesia, predominantly among Hispanic patients, it was found that the most important cause of patient anxiety was pain [20]. In another survey study, similar results were obtained regarding anxiety [21]. Finally, another study revealed that, unlike the others, fear of death is the most significant cause of patient anxiety [22]. The second cause of anxiety in the general anesthesia group was postoperative pain, while the regional anesthesia group feared needles and intervention. Nausea and vomiting were the slightest cause of anxiety in both groups. 

The main reason why the causes of anxiety in both groups differed may be that the patients were informed about the anesthesia to be applied before the consent form was signed and after randomization. The detailed information given may have reduced preoperative anxiety. However, the opposite seems possible as well. A similar study emphasized that awareness during anxiety is the leading cause of anxiety [23]. These different results between studies may arise from diversity in sociological, cultural, and local societal traditions.

When we divided the patients into sociocultural, age, and gender classes, some differences were found in the main reasons affecting anxiety. For example, in accordance with the literature, it was determined that female patients in both groups had statistically higher anxiety scores than male patients [21,24]. Another study found that the mean APAIS-A score of female patients was higher than that of men, consistent with the presented research findings, and APAIS-I scores did not differ between genders [25]. Some studies have stated that the reason for this is fluctuations in estrogen and progesterone levels [26]. In another study, the authors stated that this situation stemmed from the inability of male patients to express their weaknesses due to the perception that the male gender represents strength in some societies. Social differences can, of course, lead to differences in research results. In the validation study of the APAIS scale in Ethiopia, it was reported that the anxiety levels did not differ by gender [27].

There was no difference between the groups in terms of education level. However, it was determined that the level of anxiety increased significantly as the education level increased in both groups. Both groups found a statistically significant difference between primary school and university graduates according to the total APAIS score. Similarly, a significant difference was found between secondary school and university graduates in the regional anesthesia group. When the groups were compared in terms of subscores, it was determined that the difference was due to the “request to obtain information” subscores. However, there was no statistically significant difference between the groups in terms of “request to obtain information” scores. 

In both groups, the anxiety level of patients with anesthesia experience was found to be significantly lower. In a study, it was reported that those with the highest level of anxiety were those who were operated on for the first time, young patients, and women [28]. Studies reporting that patients’ previous surgery and anesthesia experience reduce preoperative anxiety explain this situation with the conditional learning model [29], because in the conditional learning model, the unconditioned fear stimulus must be encountered within short intervals. In the present study, both the “anxiety” and “willingness to obtain information” subscores of patients without anesthesia experience were found to be significantly higher. A study conducted in parallel with this result observed that patients’ anxiety levels decreased when information was given about the procedure [30]. A meta-analysis including 68 studies reported that the postoperative outcomes (duration of hospital stay, sedative use, recovery, and complications) of patients who received preoperative training were 20% better than those who did not [31]. In our study, the hospital stay duration was significantly shorter in patients included in the regional anesthesia group. However, complications such as delayed recovery after general anesthesia, respiratory depression due to narcotic analgesics, or possible residual neuromuscular agents may have affected the length of stay.

In addition, no relationship was found between age and anxiety scores in this study. Contrary to the results of this study, in some studies investigating preoperative anxiety, elderly patients were found to have lower anxiety levels [21,32]. The authors reported that this might be since elderly patients are more accepting of death and similar situations. Young people might also be more likely to learn about terrible health outcomes because they use the Internet more efficiently [25].

Our study compared the concerns about brachial plexus block and general anesthesia in orthopedic upper-extremity surgery and clarified the concerns in both anesthesia methods with the APAIS questionnaire. Different concerns that come to the fore according to the type of anesthesia should try to be resolve by anesthesiologists. Many studies have found that perioperative anxiety is associated with an increased need for anesthesia, postoperative pain, recovery time, and hospital stay [33,34]. To reduce anxiety, detailed information can be given about the procedure, psychological support can be provided, and anxiolytic treatments can be used. The study has some limitations. The fact that it was a single-center study may have restricted the variations in the patient population, thus limiting the effectiveness of the study. On the other hand, the study included only one type of plexus block, as regional anesthesia may have prevented us from making a general evaluation in terms of regional interventions against general anesthesia.

## 5. Conclusions

A USG-guided brachial plexus block may cause less anxiety in patients who will undergo upper-extremity orthopedic surgery. However, depending on the type of surgery and anesthesia to be applied, the degree of anxiety and the reasons for concern of the patients may vary. The APAIS questionnaire is a simple and useful method to measure patient anxiety and can be used in daily anesthesia practice, supplemented with specific anxiety-inducing questions for general and regional anesthesia. In this way, the most appropriate type of anesthesia to be applied to the patient can be decided.

## Figures and Tables

**Table 1:** Demographic data of the study participants.

	Group GA (*n* = 60)	Group BPB (*n* = 60)	*p*
Age (years)	50.53 ± 19.03	46.80 ± 11.57	0.522
**Gender**			
Female	27–45.0%	22–36.7%	0.353
Male	33–55.0%	38–63.3%	
ASA			
I	19–31.7%	12–20.0%	0.174
II	27–45.0%	37–61.7%	
III	14–23.3%	11–18.3%	
Education			
Primary school	5–8.3%	4–7.5%	0.136
Secondary school	8–13.3%	13–24.5%	
High school	35–58.3%	20–37.7%	
University	12–20.0%	16–30.2%	
Operation type			
ARIF	41–68.3%	48–80.0%	0.338
Arthroscopy	12–20.0%	8–13.3%	
Other	7–11.7%	4–6.7%	
History of anesthesia			
Yes	37–61.7%	44–73.3%	0.172
No	23–38.3%	16–26.7%	
Type of anesthesia history			
General	24–66.7%	29–65.9%	0.251
Regional	5–13.9%	11–25.0%	
Both	7–19.4%	4–9.1%	

Data are presented as mean ± standard deviation (SD) or number (%). ASA: American Society of Anesthesiologist, GA: general anesthesia, and BPB: brachial plexus block.

**Table 2:** Comparison of anxiety scores, pain intensity, and postoperative data of the study groups.

	Group GA (*n* = 60)	Group BPB (*n* = 60)	*p*
APAIS scale			
Anxiety score	12 (8–17)	9 (5–14)	**0.007**
Information wish score	4 (2–7)	4 (2–7)	0.429
Total score	17 (13–23)	13 (7–20)	**0.021**
Numeric Rating Scale			
1 h	8 (6–10)	0 (0–3)	**<0.001**
8 h	5 (2–7)	2 (0–4)	**<0.001**
16 h	5 (2–7)	3 (0–6)	**0.044**
24 h	4 (2–6)	4 (2–7)	0.750
Duration of surgery	110.93 ± 47.81	103.20 ± 61.11	0.702
LOS	3 (1–5)	2 (1–4)	**0.028**
Tramadol consumption	176.00 ± 71.60	62.00 ± 40.03	**<0.001**

Data are presented as mean ± standard deviation (SD) or median (IQR). APAIS: Amsterdam Preoperative Anxiety and Information Scale, GA: general anesthesia, BPB: brachial plexus block, and LOS: length of hospital stay. *p* values that are in bold are statistically significant.

**Table 3:** Association of anesthesia-related concern subscores and total scores.

	Group GA (*n* = 60)	Group BPB (*n* = 60)	*p*
Fear of death	4 (2–5)	1 (1–3)	**<0.001**
Experience of anesthesiologist	3 (1–5)	3 (1–4)	0.129
Not being able to wake up after surgery	4 (2–5)	2 (1–4)	**0.007**
Feeling pain during the operation	3 (1–5)	2 (2–5)	**0.004**
Postoperative pain	4 (1–5)	2 (1–4)	**0.010**
Waking up in middle of surgery	3 (2–4)	1 (1–3)	**<0.001**
Postoperative nausea and vomiting	2 (1–3)	2 (1–2)	0.586
Behavior of anesthesiologist	2 (1–3)	2 (1–4)	0.700
To be admitted to intensive care	3 (2–5)	3 (1–3)	0.895
Sleeping for a long time after surgery	2 (1–2)	2 (1–2)	0.464
Fear of needles and interventions	2 (2–5)	4 (2–5)	**0.021**

Data are presented as median (IQR). GA: general anesthesia, BPB: brachial plexus block, and LOS: length of hospital stay. *p* values that are in bold are statistically significant.

**Table 4:** Association of demographic and anesthesia history and APAIS scores.

	Group GA (*n* = 60)			Group BPB (*n* = 60)		
	Anxiety Score	Information Score	Total Score	Anxiety Score	Information Score	Total Score
Age (years)						
r	−0.161	−0.249	−0.197	−0.188	0.194	−0.066
*p*	0.566	0.371	0.481	0.503	0.488	0.814
Gender						
Male	8 (4–18)	3 (2–8)	11 (6–29)	6 (4–15)	3 (2–7)	7 (6–22)
Female	17 (6–20)	7 (4–10)	22 (6–30)	14 (6–16)	6 (4–10)	17 (10–23)
*p*	**0.001**	**0.003**	**0.001**	**0.009**	0.079	**0.015**
**Education**						
Primary School	8 (4–13)	3 (2–4)	11 (6–17)	4 (4–14)	3 (2–10)	6 (6–18)
Secondary School	6 (4–10)	3 (2–4)	10 (6–14)	6 (5–18)	3 (2–8)	8 (7–26)
High School	13 (6–19)	4 (2–9)	16 (8–27)	9 (6–17)	4 (2–9)	12 (11–24)
University	17 (7–20)	7 (3–10)	24 (8–30)	11 (4–16)	5 (2–10)	15 (12–23)
*p*	**0.011**	0.076	**0.041**	0.738	0.232	**0.011**
History of Anesthesia						
Yes	9 (4–16)	4 (2–7)	14 (6–23)	8 (4–15)	3 (2–8)	10 (6–22)
No	14 (4–20)	7 (4–10)	21 (6–30)	10 (5–16)	7 (3–10)	17 (8–23)
*p*	0.236	**0.004**	**0.036**	0.293	**0.029**	**0.017**
Type of Anesthesia History						
General Anesthesia	13 (4–16)	4 (2–10)	18 (7–21)	11 (4–18)	5 (2–10)	12 (6–26)
Regional Anesthesia	9 (7–18)	4 (2–9)	14 (6–30)	8 (6–20)	3 (2–10)	15 (6–30)
Both	8 (4–20)	3 (2–10)	11 (6–26)	9 (6–20)	4 (2–10)	14 (8–27)
*p*	0.109	0.238	0.151	0.149	0.698	0.307

Data are presented as median (IQR). GA: general anesthesia, BPB: brachial plexus block, and LOS: length of hospital stay. *p* values that are in bold are statistically significant.

## Data Availability

The data presented in this study are available on request from the corresponding author. The data are not publicly available due to privacy reasons.
